# Correction: Using whole-genome sequence data to examine the epidemiology of Salmonella, Escherichia coli and associated antimicrobial resistance in raccoons (Procyon lotor), swine manure pits, and soil samples on swine farms in southern Ontario, Canada

**DOI:** 10.1371/journal.pone.0308217

**Published:** 2024-07-29

**Authors:** Nadine A. Vogt, Benjamin M. Hetman, David L. Pearl, Adam A. Vogt, Richard J. Reid-Smith, E. Jane Parmley, Nicol Janecko, Amrita Bharat, Michael R. Mulvey, Nicole Ricker, Kristin J. Bondo, Samantha E. Allen, Claire M. Jardine

The bioproject number in column A of S1 File should have been “PRJNA745182” for all 255 rows. Please view the correct S1 File below.

## Supporting information

S1 File (XLSX)
