# Supplementary material for: Using whole-genome sequence data to examine the epidemiology of Salmonella, Escherichia coli and associated antimicrobial resistance in raccoons (Procyon lotor), swine manure pits, and soil samples on swine farms in southern Ontario, Canada
Source: PLoS One. 2021 Nov 18;16(11):e0260234. doi: 10.1371/journal.pone.0260234 (PMC8601536; doi:10.1371/journal.pone.0260234)
Supplement: S1 Table — (DOCX) [file pone.0260234.s002.docx]

Supplementary Table S1: Multi-locus sequence types identified using whole-genome sequencing data from antimicrobial resistant *Escherichia coli* isolates obtained from raccoons, swine manure pits, and soil samples on swine farms in southern Ontario, Canada 2011–2013 (n=96)

| **Sequence type** | **Count (%)** |
| --- | --- |
| 10^a^ | 11 (11.5%) |
| 101 | 9 (8.7%) |
| 58 | 5 (5.2%) |
| 542 | 4 (3.9%) |
| 1633 | 4 (3.9%) |
| 155 | 3 (2.9%) |
| 34 | 3 (2.9%) |

^a^ This sequence type has been implicated in human uropathogenic *Escherichia coli* (UPEC) infections. Other sequence types frequently associated with UPEC infections were not identified here (e.g., ST131, ST96, ST73, ST127, ST140).

*Sequence types identified in fewer than 3 samples were: 43, 898, 349, 345, 295, 3714, 7324, 1844, 1721, 641, 1727, 847, 1771, 48, 547, 1406, 106, 973, 871, 218, 117, 1324, 2178, 9982, 1086, 2077, 206, 3531, 388, 716, 6777, 6975, 4085, 2562, 1079, 212, 602, 5614, 4429, 1112, 362, 1304.
