# Supplementary material for: Using whole-genome sequence data to examine the epidemiology of Salmonella, Escherichia coli and associated antimicrobial resistance in raccoons (Procyon lotor), swine manure pits, and soil samples on swine farms in southern Ontario, Canada
Source: PLoS One. 2021 Nov 18;16(11):e0260234. doi: 10.1371/journal.pone.0260234 (PMC8601536; doi:10.1371/journal.pone.0260234)
Supplement: S2 Table — (DOCX) [file pone.0260234.s003.docx]

Supplementary Table S2: Serovars identified using whole-genome sequencing data from phenotypically resistant *Escherichia coli* isolates obtained from raccoons, swine manure pits, and soil samples on swine farms in southern Ontario, Canada 2011–2013 (n=96)

| **Serovar** | **Count (%)** |
| --- | --- |
| O82:H8 | 6 (6.3%) |
| O6:H10 | 2 (2.1%) |
| O108:H2 | 2 (1.9%) |
| O8:H49 | 2 (1.9%) |
| O8:H25 | 2 (1.9%) |
| O83:H14 | 2 (1.9%) |
| O62:H30 | 2 (1.9%) |
| O86:H51 | 2 (1.9%) |
| O109:H45 | 2 (1.9%) |
| O42/O28:H21 | 2 (1.9%) |
| O46/O8/O134:H21 | 2 (1.9%) |
| O96:H5 | 2 (1.9%) |
| O166:H15 | 2 (1.9%) |

*****Additional serovars not listed here were identified in fewer than two samples or could not be typed (n=24). Serogroups responsible for the majority of Shiga-toxin producing *E. coli* infections in humans (i.e., O157, O26, O91, O45, O103, O111, O121, O145) were not identified here.
