# Supplementary material for: Using whole-genome sequence data to examine the epidemiology of Salmonella, Escherichia coli and associated antimicrobial resistance in raccoons (Procyon lotor), swine manure pits, and soil samples on swine farms in southern Ontario, Canada
Source: PLoS One. 2021 Nov 18;16(11):e0260234. doi: 10.1371/journal.pone.0260234 (PMC8601536; doi:10.1371/journal.pone.0260234)
Supplement: S4 Table — (DOCX) [file pone.0260234.s005.docx]

Supplementary Table S4: Contrasts from univariable logistic regression models^a,b^  (Table 7) assessing the statistically significant associations between farm location, source type, and the occurrence of select antimicrobial resistance genes and plasmid incompatibility (Inc) types in *Escherichia coli* isolates from raccoons, swine manure pits, and soil samples on swine farms in southern Ontario, Canada 2011-2013 (n=96)

|  | **IncI1(alpha)^a^** | |  | **IncFIB(AP001918)^b^** | | **sul2^b^** | |
| --- | --- | --- | --- | --- | --- | --- | --- |
| **Contrast** | **OR (95%CI)** | ***p*-value** | **Contrast** | **OR (95%CI)** | ***p*-value** | **OR (95%CI)** | ***p*-value** |
| Farm 8 vs. 7 | 0.21* (0–1.58) | 0.155 | Raccoon vs. soil | 1.57 (0.54–4.57) | 0.409 | 1.71 (0.51–5.71) | 0.380 |
| Farm 9 vs. 7 | 0.56 (0.08–3.03) | 0.490 | – | – | – | – | – |
| Farm 10 vs. 7 | 0.10* (0–0.70) | 0.011 | – | – | – | – | – |
| Farm 9 vs. 8 | 2.48* (0.24–∞) | 0.273 | – | – | – | – | – |
| Farm 10 vs. 8 | 1 (0–∞) | – | – | – | – | – | – |
| Farm 10 vs. 9 | 0.19* (0–1.87) | 0.081 | – | – | – | – | – |

* Median unbiased estimates.

^a^ Exact logistic regression model.

^b^ Ordinary logistic regression model.
